# Enhanced Skin Permeation of Estradiol by Dimethyl Sulfoxide Containing Transdermal Patches

**DOI:** 10.3390/pharmaceutics13030320

**Published:** 2021-03-01

**Authors:** Anna Otterbach, Alf Lamprecht

**Affiliations:** 1 Department of Pharmaceutics, Institute of Pharmacy, University of Bonn, Gerhard-Domagk-Str. 3, 53121 Bonn, Germany; annaotterbach@uni-bonn.de; 2 PEPITE EA4267, University of Burgundy/Franche-Comté, 25000 Besançon, France

**Keywords:** transdermal patch, dimethyl sulfoxide, enhanced skin permeation, estradiol permeation, recrystallization inhibition

## Abstract

Dimethyl sulfoxide is a well-known and widely used dermal penetration enhancer. Its incorporation in transdermal patches would be highly desirable; however, due to its volatility this is extremely challenging. Here, we report on the feasibility of a dimethyl sulfoxide (DMSO) containing transdermal system containing estradiol as a model compound. Transdermal patches were prepared from duro-tak^®^ 387-2510 containing various DMSO concentrations at different drying temperatures. The resulting patches were analyzed for DMSO content, estradiol and DMSO release, estradiol and DMSO permeation through excised porcine skin, and recrystallization during stability testing. Drying conditions in the range of 35° to 40° allowed a complete polymer solvents removal while retaining significant amounts of DMSO (≤10 mg/patch). Estradiol skin permeation increased 4-fold (Jss = 4.12 µg/cm^−2^·h^−1^) compared to DMSO-negative control (Jss = 1.1 ± 0.2 µg/cm^−2^·h^−1^). As additional benefit, estradiol recrystallization was inhibited by DMSO at even lowest solvent concentrations. Storage stability was limited to 6 months at 25 °C with a surprising discrepancy between DMSO content (significantly lower) and flux (not significantly different). Although the technical feasibility range is relatively narrow, such DMSO-containing matrix-type patches are able to significantly enhance drug permeation through the skin while ameliorating the product stability against recrystallization.

## 1. Introduction

The skin is the largest organ of the body and although it functions as an effective barrier, the skin represents an attractive option for the administration of drugs for their systemic availability. Drug delivery across the skin is an essential alternative over, e.g., the oral drug administration, because it offers a number of advantages over other routes of delivery, including avoiding first-pass metabolism, reducing peak plasma concentrations, the potential of delivering at controlled rates, and increasing patient compliance [1]. Since transdermal administration is not suitable for all drugs or therapeutic indications, it requires a detailed study of the physicochemical and biological properties of the drugs in question. A successful drug molecule for passive transdermal delivery is preferentially nonionic, lipophilic, and effective at low doses, and has a low molecular weight (<500 Da) [2,3].

Especially transdermal therapeutic systems (TTS) focus on the beforementioned advantages of the delivery through the skin, and can provide a relatively constant plasma level of the active over a longer time period. ß-Estradiol (E2) is such a drug candidate which is commonly administered via the skin, because this application allows to meet the required therapeutic regimen for estradiol by a continuous uniform systemic drug delivery up to one week [4]. E2 is used for menopausal hormone replacement therapy to prevent osteoporotic fractures and to reduce the breast cancer incidence [5]. In this therapeutic context, constant plasma levels of E2 are most desirable while peaks and troughs, as they typically occur during an oral administration, should be avoided. According to these properties, E2 was used as model drug to evaluate the feasibility of this study.

When formulated as transdermal patch, passive diffusion is the driving force to promote the distribution of the drug from the transdermal patches into and through the skin [6]. Accordingly, the drug is typically formulated in a saturated to supersaturated state within the transdermal patches in order to reach maximum drug gradients. Such systems, however, are thermodynamically unstable and have a high risk of recrystallization of drugs, especially during storage, which often occurs with drugs with limited solubility in the matrix of the carrier system [7]. To overcome this problem, diverse formulation approaches have been tested, typically based on the use of crystallization inhibiting excipients to stabilize the supersaturated condition in transdermal patch formulation [7]. Among them, mainly polymers have been tested, such as polyvinylpyrrolidones [8] or methacrylic acid copolymers [9]. Although toxicity after permeation is a general issue with organic solvents, they are missing on this list due to their high volatility, which essentially risks their premature elimination by evaporation during the formulation steps.

However, even in supersaturated state, drug candidates can still exhibit therapeutically insufficient drug absorption through the skin without the additional use of permeation enhancing components. Accordingly, to enhance the drug delivery across the skin barrier, dermal drug formulations typically comprise excipients that allow increased drug permeation. Within an extremely large choice of chemically completely differing compounds, organic solvents were found to enhance the skin permeability nearly half a century ago [10]. Among them, dimethyl sulfoxide (DMSO) has become somewhat a first-choice, as an organic solvent that exhibits significant permeation enhancing potential, combined with its versatile solvent properties and the relatively low vapor pressure. Besides, its toxicity is relatively limited compared to other solvents with this potential [11,12]. The molecule consists of a hydrophilic sulfoxide group and two hydrophobic methyl groups, which provides an amphiphilic character. Due to its structural features, DMSO interacts with cell membranes, resulting in an enhanced permeation of hydrophilic, as well as hydrophobic drugs. The effect on cell membranes depends on the concentration of DMSO, from an increased bilayer-fluidity at lower concentrations, through to the formation of water pores and the extraction of lipid molecules at higher concentrations [13,14].

The ‘drug-in-adhesive’ type patches (DIA) consist of a matrix polymer, which is typically dissolved together with the active in an organic solvent mixture to allow their formulation as a liquid and castable drug-in-adhesive mass [1]. However, by the end of the production cycle, those solvents must be eliminated from the liquid mass to enable the curing of the drug-containing adhesive mix. Insufficient solvent evaporation would inhibit the formation of the solid DIA-structure on one hand, and create toxicological issues on the other. Accordingly, it is a major challenge to incorporate a volatile permeation enhancer that could increase permeated drug amount and simultaneously stabilize the supersaturated patch matrix from drug recrystallization while evaporating the matrix polymer solvents to solidify the patch. Here, we report on the feasibility of a DMSO-containing DIA based on duro-tak^®^ and to which extent the flux of E2 across the skin can be enhanced. Preliminary stability tests indicate a surprisingly long storage period at room temperature still exhibiting constant E2 flux across the skin.

## 2. Material and Methods

### 2.1. Materials

DMSO was supplied by VWR (Darmstadt, Germany). ß-Estradiol (E2), 1,3-dimethyl-2-imidazolidinone, and γ-Cyclodextrin were obtained by TCI Chemicals (Eschborn, Germany). Duro-Tak^®^ 387-2510 (DT) is an acrylate polymer that was used as adhesive matrix of the DIA. Solid content of the viscous gel as obtained from the supplier was 41.5%; it contained ethylacetate and hexane and was a gift from Henkel (Waalwijk, The Netherlands). Ethylacetate (EA), acetonitrile, sodium lauryl sulphate (SLS), methanol, and acetone were purchased from Merck (Darmstadt, Germany). Backing liner (3M^TM^ Scotchpak^TM^) and release liner (3M^TM^ Scotchpak^TM^) were obtained as a gift from 3M^TM^. All chemicals were of analytical grade. Pig ears were obtained from a local abattoir immediately post-sacrifice and prior to steam cleaning (Bonn, Germany).

### 2.2. Preparation and Evaluation of DIA

Patches were prepared by solvent evaporation technique. Twenty milligrams of E2 was dissolved in 1 g DMSO and mixed with 0.55 g EA and 1 g DT. A control batch without DMSO was prepared by dissolving E2 directly in EA and mixing the solution with DT. Viscous solutions were cast on a drug-impermeable and occlusive backing liner, using a film applicator (Multicator 411, Erichsen, Hemer, Germany) and spread to a thickness of 460 µm. Subsequently, the laminates were dried for different periods of time at different temperatures in a forced convection oven. The dried laminates were covered with a fluoropolymer-coated release liner and cut into circular DIAs with a diameter of 1.5 cm. The drying process at 20 °C, 30 °C, 35 °C, 40 °C, and 50 °C was monitored gravimetrically and via gas chromatography (GC) to evaluate the kinetics and the ideal conditions of drying. Drug load of the patches was determined by dissolving each patch (before or after permeation experiments) in 5 mL acetone and adding 10 mL of 2.5 g/L SLS solution to precipitate the polymer. The mixture was centrifuged at 15,000 rpm at 20 °C (RCF = 21.380); subsequently, the supernatant was assayed by the high-performance liquid chromatography (HPLC) method described below (recovery rate: 98.2 ± 5.3%). The residue was completely dried and weighed to determine the polymer mass.

Although drying at 50 °C for 2 h led to complete solvent removal as well as DMSO loss, DMSO-negative control patches were standardly dried at 50 °C for 24 h and used for comparative studies thereafter.

### 2.3. High-Performance Liquid Chromatography (HPLC) of E2

A Waters (Milford, MA, USA) 2695 Separations Module combined with a Waters 996 Photodiode Array Detector was used. Analysis were performed with a reversed-phase C18 column (LiChrospher 100 RP 18-5µ; 250 mm length; 4.6 mm inner diameter, 5 µm particle size) using a modified method from Ph. Eur 9.0 [15]. The mobile phase consisted of water and Acetonitrile (50/50, *v*/*v*) running isocratic at 1 mL/min; column temperature was kept at 25 °C. Detection was performed at 220 nm (LOQ = 100 ng, LOD = 40).

### 2.4. Residual Solvent Analysis

Measurements were performed using a modified static-headspace method according to Ph. Eur 9.0 [16]. The amount of Hexane, EA and DMSO was determined with a Focus GC Gas Chromatograph, equipped with a flame ionization detector (FID) and TriPlus RHS Autosampler (ThermoFisher Scientific, Dreieich, Germany). The initial adhesive mass, respectively, the DIAs, were dissolved in 1.3-dimethyl-2-imidazolidinone and transferred into closed glass vials. The headspace oven was maintained at 100 °C, and the sample was heated and agitated for 25 min; afterwards, a sample volume of 1 mL was withdrawn and injected. An FS-CS-624 column (30 m length, 0.32 mm ID, Chromatography Service, Langerwehe, Germany) was used. N_2_ was used as carrier gas with a flow of 2.0 mL/min, FID kept at 240 °C. The oven program for residual solvent analysis of the patches started at 70 °C and heated up to 130 °C at 30 °C/min. The final heating phase (60 °C/min) resulted in a temperature of 220 °C; this temperature was kept constant for 1.5 min. The limits of quantification were 4.5 ng for EA (LOD: 2 ng), 3.3 ng for hexane (LOD: 1 ng), and 5.5 µg for DMSO (LOD: 2 µg).

Aqueous samples received from permeation experiments were diluted with methanol and analyzed via liquid injection. An AS-JXR column (15 m length, Altmann Analytik, München, Germany) was used. The oven program started at 80 °C, followed by heating up to 140 °C at 50 °C/min. The final temperature of 220 °C (90 °C/min) was kept constant for 1 min.

### 2.5. In Vitro Drug Release

The release of E2 from DIAs was analyzed with a modified franz diffusion cell apparatus equipped with jacketed franz-cells and a heater/circulator system (SES Analysensysteme, Bechenheim, Germany). The release liner was removed from the DIA. Afterwards, the patch (diameter 1.15 cm) was placed and fixed over the circular orifice (diameter: 1.15 cm), with the adhesive-side facing towards receiver compartment ensuring the absence of air bubbles between patch and receptor fluid. The acceptor compartment was filled with PBS, phosphate buffered saline, (V = 8 mL, pH 7.4) and 0.5% γ-cyclodextrin was added to increase the solubility of E2 in the acceptor phase (0.1 mg/mL) [17]. The acceptor solution was continuously stirred and kept at 32 °C, samples were withdrawn at predetermined time points, analyzed by HPLC and replaced with fresh acceptor solution. Experiments were performed in triplicate. The drug release kinetics were obtained by fitting the in-vitro release data according to Korsmeyer–Peppas (R^2^ ≥ 0.9) [18].

### 2.6. In Vitro Skin Permeation Studies

Pig ears were gently cleaned and excised upon reception. The skin was separated from the porcine ear by carefully removing the connecting tissues with a surgical scalpel. After removing most of the subcutaneous fat, the skin was placed between two even plates, which were clamped together and cooled to −20 °C in a freezer. The frozen skin was removed from the freezer and the plate covering the stratum corneum taken off. A dermatome was used to cut skin strips with a thickness of 1 mm, the obtained strips were cut to circular disks using a die punch [19,20]. Subsequently, the integrity of the samples was assessed by visual inspection and by checking the tightness of the skins mounted onto jacketed franz diffusion cells (SES Analysensysteme, Bechenheim, Germany). Skin samples were stored at −20 °C and thawed at room temperature before use.

The prepared skin was placed between the donor and acceptor compartment of the cells, a pinch clamp secured both parts of the cells [19,21]. The acceptor compartment was filled entirely with PBS containing 0.5% γ -cyclodextrin. Initially, 2 mL of the acceptor solution were placed on top of the skin. The system was maintained at 32 ± 0.5 °C [19] and continuously stirred with a magnetic bead for 1 h to equilibrate the skin. Stirring was performed to maintain a constant hydrodynamic flow of the acceptor solution. After equilibration, the top side of the skin was dried and a DIA was placed upon the skin surface. After 1, 2, 4, 6, 9, 24, and 28 h, 2 mL of acceptor fluid were withdrawn through the sampling port and replaced with fresh acceptor solution, avoiding any air bubble under the skin.

Drug concentrations were determined by HPLC, and the amount of DMSO was determined by GC. Experiments were performed in triplicate. Flux J_SS_ [µg/h·cm^2^] is constant within the steady-state period and was calculated by linear regression analysis from the slope of the plot of cumulative E2/DMSO permeated per cm^2^ of skin (dQ) against the time (dt). The lag-time is the intercept of J_SS_ with the time axis.

Following the permeation experiments, skin samples were cut into thin horizontal slices with a cryo-microtome (SLEE medical, Mainz, Germany). Acetone and methanol were added to the slices to extract the penetrated drug amount. The mixture was homogenized and centrifuged; the supernatant was assayed via HPLC.

### 2.7. X-ray Diffraction (XRD)

Pure drug powder and the prepared patches were assessed for crystallinity using X-ray diffraction. XRD measurements were performed in reflection mode (X’Pert MRD Pro, PANalytical, Almelo, Netherlands) with an X’Celerator detector and nickel filtered CuK α1 radiation (λ = 1.54 Å) at 45 kV and 40 mA in the angular scan range from 4° to 45° 2ϴ at a scan speed of 0.017°/s. Patches were mounted on sample holders (15 mm) without the release liner, matrix-site facing upwards. Data evaluation was performed with X’Pert High Score version 2.2.

### 2.8. Stability Evaluation

Stability studies were performed over 6 months at different conditions (fridge: 2–8 °C or climate chamber: 25 °C/60% RH). Sealed (PET-AL-composite foil, Medewo, Germany) samples were stored at both conditions. Additionally, unsealed samples were stored at 25 °C/60% RH. After 1, 2, 3 and 6 months, stability samples were tested for residual DMSO amount. After 3 and 6 months, samples were additionally tested for in vitro permeation and XRPD measurements were performed.

### 2.9. Statistics

All results were expressed as mean values ± SD. Statistical analysis were performed by GraphPad software (San Diego, CA, USA) using analysis of variance test (ANOVA), followed by significance analysis with Sidaks’s multiple comparison test. Differences were considered significant at levels of * *p* < 0.05, ** *p* < 0.01 and *** *p* < 0.001. Surface plots have been generated using OriginPro 8G (OriginLab Corporation, Northampton, MA, USA).

## 3. Results

### 3.1. Patch Preparation and Evaluation

**Figure 1:**
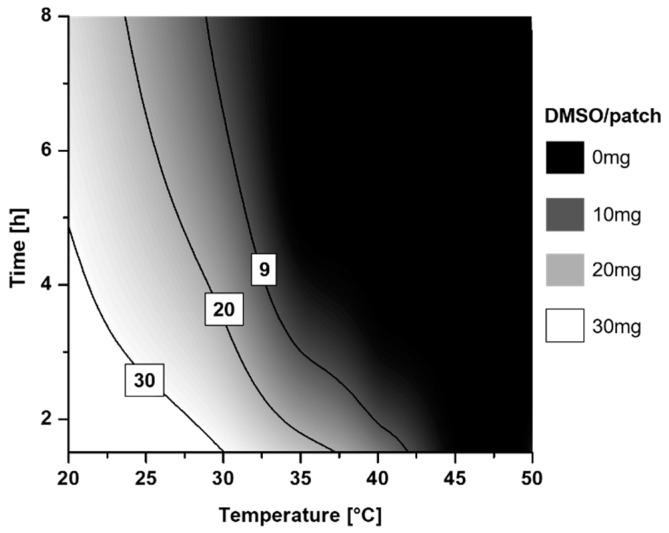
Contour plot of the effect of drying time and temperature on the residual amount of DMSO/patch. The line with the value 9 displays the maximum amount of DMSO (9 mg) which can be incorporated to produce a suitable transdermal patch.

**Figure 2:**
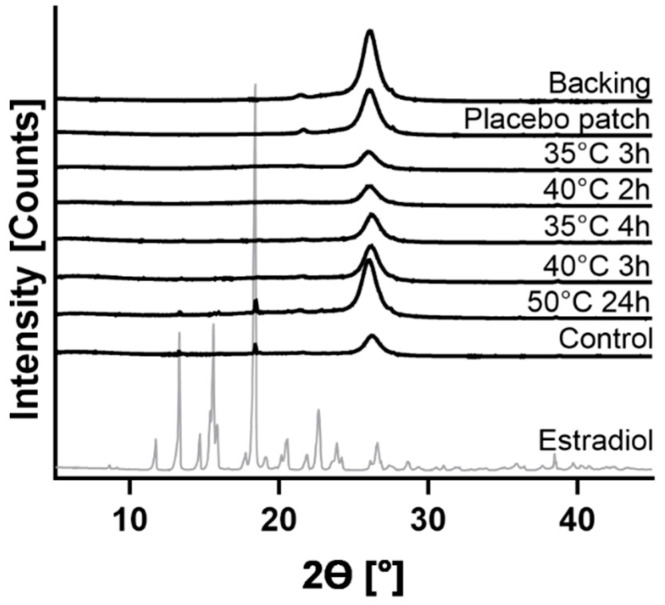
**The** XRD patterns of pure E2 and the prepared patches. The placebo patch was prepared without DMSO and E2 (consisting of DT), and the control patch was prepared without DMSO (consisting of E2 + DT).

To eliminate the organic solvents ethyl acetate and n-hexane to a level of unquantifiable amounts, a minimum drying temperature of 30 °C combined with a minimum duration of 4 h was found necessary. Accordingly, an attempt of drying at 20 °C for 4 h led to significant residual ethyl acetate content. Generally, it was found relatively difficult to retain DMSO in the patches being a total solvent, and simultaneously DMSO evaporation was observed at drying conditions that were relatively similar, i.e., after 2 h of drying at 50 °C, after 4 h at 40 °C and after 16 h at 30 °C (Figure 1, Table 1). Figure 1 illustrates the drying conditions and the subsequent DMSO content per patch, while threshold values of around 10 mg of DMSO/patch were the maximum of DMSO which could be incorporated before the formulations turned into a wet and unusable gel mass. However, patches with drying conditions at 35 °C (3 h and 4 h) and 40 °C (2 h and 3 h) had sufficiently high energy input during drying in order to obtain solid patch formulations. E2 recrystallization was observed only in patches prepared at 50 °C for 24 h, which had DMSO in the initial mixture and in the DMSO-negative controls (Figure 2). Peak heights were 3.1% resp. 4.7% compared to the reference peak of pure E2 at 18.39°. All other patterns showed the absence of detectable peaks at 18.4°.

### 3.2. Drug Release and Permeation Behavior

**Figure 3:**
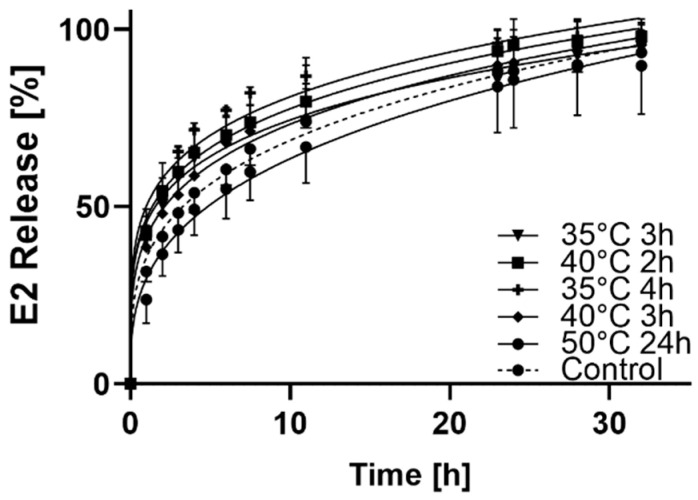
In vitro release profile in phosphate buffered saline (PBS, pH 7.4) at 32 °C.

In general, estradiol-release curves of patches with different amounts of DMSO are comparable (Figure 3). The total amount of E2 was released within 32 h from 40 °C, 2 h. The patches prepared at 35 °C, 4 h and 35 °C, 3 h showed 80% and drug release for the remaining patches was 70%–75%.

**Figure 4:**
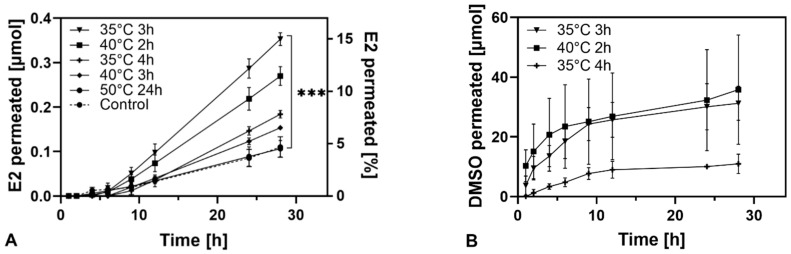
Permeation profiles of E2 (**A**) and DMSO (**B**) across pig skin at 32 °C. Percental permeated amount of DMSO: 35 °C, 4 h: 11.5 ± 0.7; 40 °C, 2 h: 21.8 ± 1.9; 35 °C, 3 h: 65.4 ± 3.1 (mean ± SD). *** Indicates significant differences (*p* < 0.001). DMSO permeation profiles showed, in contrast to E2, a neglectable lag time (Figure 4B) and also visibly permeated faster through the skin than the drug. The permeated amount of DMSO from 40 °C, 3 h was not detectable in the current experimental setting. Besides, the permeated DMSO amount reached a slowly increasing plateau after 6–9 h with all respective patches. Again, similar to E2, the flux and permeated DMSO amount increased with increasing initial DMSO content of the patch (Figure 5B).

While release profiles of the different formulations were closely related, E2 skin permeation showed significant differences between the patch types (Figure 4A) where the permeated E2 amount correlated to the DMSO amount after preparation. After 28 h, the cumulative drug amount of the formulation containing the highest amount of DMSO (35 °C, 3 h) was four times higher than of 50 °C, 24 h (Table 2). The highest mean dose delivered was 82 µg/24 h (35 °C, 3 h), the lowest for 50 °C, 24 h and control were 26 µg/24 h and 24 µg/24 h, respectively. Lag times increased with increasing DMSO content in the patches.

**Figure 5:**
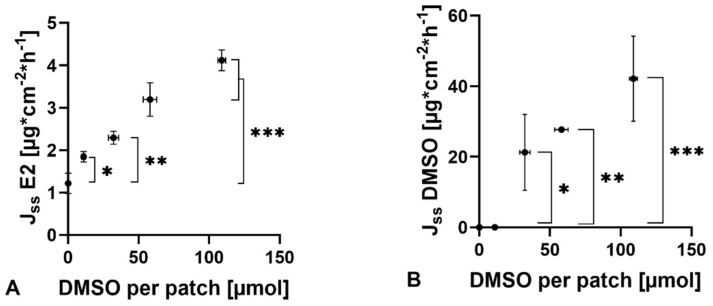

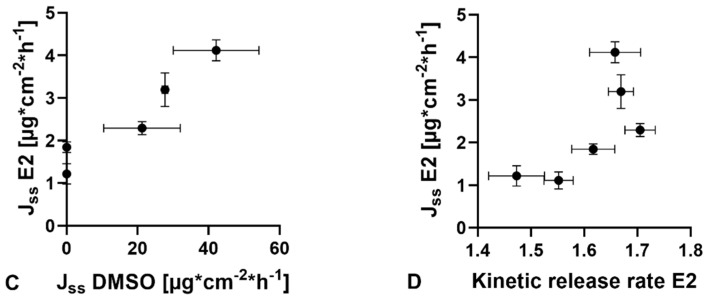
(**A**) Correlation between steady-state flux (J_ss_ E2) of estradiol and DMSO per patch (R^2^ = 0.972). (**B**) Correlation between steady-state flux of DMSO (J_ss_ DMSO) and DMSO per patch (R^2^ = 0.924). (**C**) Correlation between steady-state flux of estradiol (J_ss_ E2) and steady-state flux of DMSO (Jss DMSO) (R^2^ = 0.884). (**D**) Correlation between kinetic release rate of estradiol (J_ss_ E2) and steady-state flux of estradiol (J_ss_ E2) (R^2^ ≤ 0.5). * Indicates significant differences (*p* < 0.05), ** indicates significant differences (*p* < 0.01) and *** indicates significant differences (*p* < 0.001).

Linear correlations were found between DMSO content in the patch and drug flux J_ss_ E2, with lowest flux values (1.22 ± 0.24 µg/cm^−2^·h^−1^) for the patches containing no DMSO (50 °C, 24 h) (Figure 5A). J_ss_ E2 increases proportionally to the amount of permeation enhancer to a maximum of 4.12 ± 0.25 µg/cm^−2^·h^−1^ for 35 °C, 3 h. Based on these data, the presence of DMSO in the patch improved drug permeation nearly 4-fold. Additionally, the drug flux J_ss_ E2 increases proportionally to the flux of DMSO (Figure 5C). There was no correlation found between permeation and release behavior of E2 (Figure 5D).

**Figure 6:**
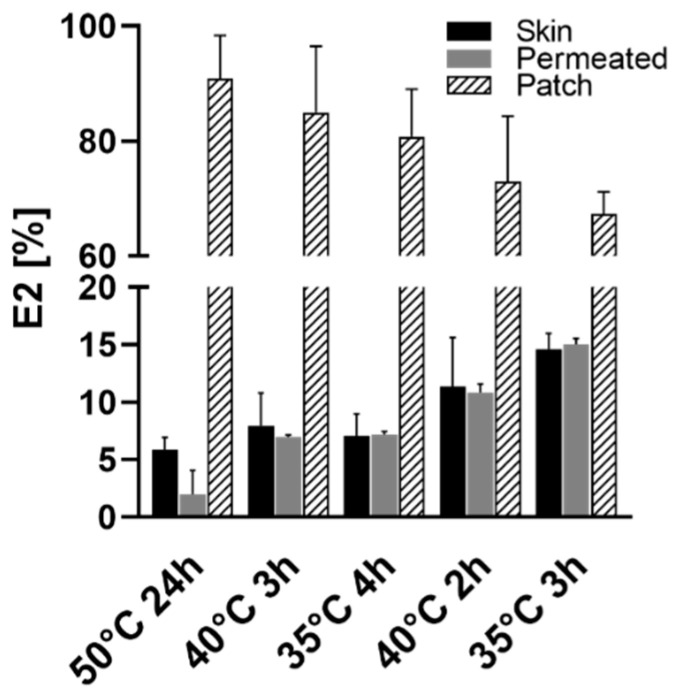
Distribution of E2 after 28 h permeation experiments in skin and patch and through permeation into the acceptor compartment of Franz cells.

The E2 distribution in the patch, skin and acceptor compartment differed significantly between the formulations (Figure 6). An increasing DMSO content per patch was associated with higher E2 amounts recovered from acceptor phase and extracted from the skin layer (35 °C, 3 h > 40 °C, 2 h > 35 °C, 4 h > 40 °C, 3 h > 50 °C, 24 h). The E2 concentrations were at similar levels when comparing skin and acceptor phase with the exception for the patch containing no DMSO (50 °C, 24 h).

### 3.3. Storage Stability

**Figure 7:**
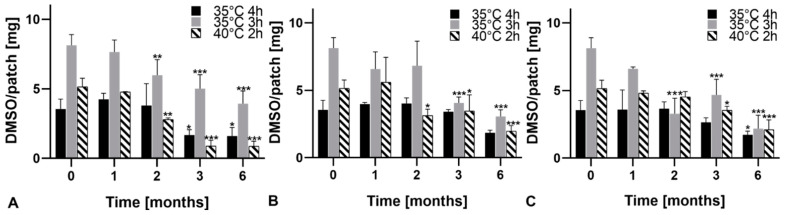
DMSO/patch at different storage conditions over a period of six months. (**A**) 2–8 °C (sealed), (**B**) 25 °C/60% RH (sealed), (**C**) 25 °C/60% RH (unsealed). Residual amounts after one, two, three and six months were compared to the initial amount at t = 0. * Indicates significant differences (*p* < 0.05), ** indicates significant differences (*p* < 0.01) and *** indicates significant differences (*p* < 0.001).

**Figure 8:**
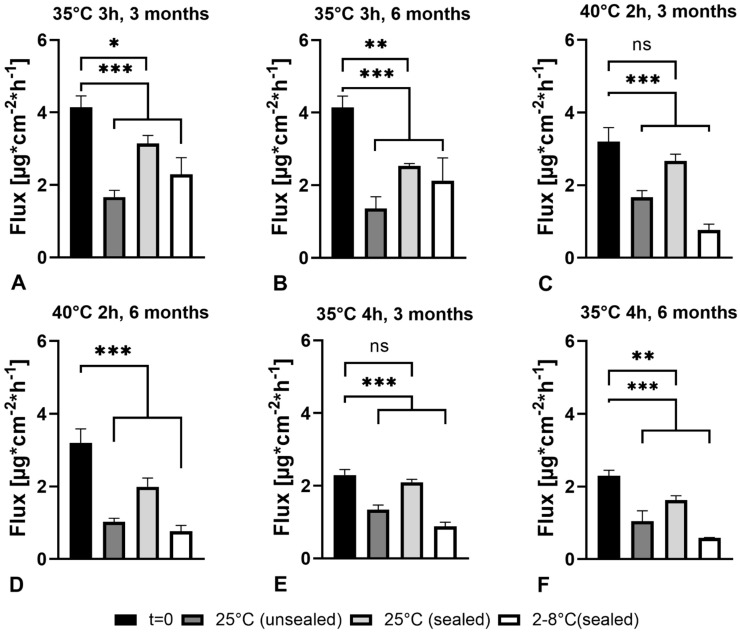
E2 permeation of 35 °C, 3h after three (**A**) and six months (**B**), 40 °C, 2h after three (**C**) and six months (**D**) and of 35 °C, 4h after three (**E**) and six months (**F**) of storage. Flux values after three and six months were compared to the initial value at t = 0. * Indicates significant differences (*p* < 0.05), ** indicates significant differences (*p* < 0.01) and *** indicates significant differences (*p* < 0.001).

The absence of recrystallization after six months for all DMSO-based formulations regardless of the storage conditions was confirmed by XRPD (data not shown). In terms of DMSO loss, patches revealed to be stable during the first month under all test conditions as DMSO-content did not differ significantly (Figure 7). The benefit of low temperatures was minor as during longer periods, significant DMSO loss occurred of all formulations when stored at 4 °C (sealed, Figure 7A). The slightest change in DMSO content was found when patches were stored sealed at 25 °C/60% RH (Figure 7B). Generally, a more distinct reduction of DMSO content in patches was found for those with the initially highest amount (35 °C, 3 h), the lowest loss for those with the lower initial DMSO content. Accordingly, after three months at 25 °C/60% RH (sealed), 40 °C, 2 h and 35 °C, 4 h patches still did not show statistically significant DMSO losses. Generally, the DMSO loss during storage translated into lower E2 permeation through the skin. While six months storage under any storage conditions led to a significant lowering of E2 permeation, E2 permeation did not significantly change in patches 40 °C, 2 h and 35 °C, 4 h after three months (Figure 8).

## 4. Discussion

Although the volatility of DMSO was surely the major formulation hurdle, this study nonetheless affirmed the feasibility of producing transdermal patches with sufficiently high amounts of DMSO in order to increase drug permeation through the skin. Nevertheless, the production process would probably need to be further optimized, especially scaled up in a continuous production. The formulation could further be improved by creating a matrix, which would sufficiently retain DMSO. This may be achieved by increasing the viscosity of the initial adhesive mass or by changing the polymer type.

Earlier studies tested transdermal permeation of a variety of drugs combined with different permeation enhancers including DMSO and could not improve drug permeation; this is surely related to the fact that drying was performed at 60/65 °C [22,23]. Consequently, the authors explained the lack of permeation enhancement with an insufficient DMSO concentration, quoting an enhancing effect with concentrations above 50%. However, it was possible to significantly enhance the permeation of Ketorolac with a concentration of 5% DMSO in a transdermal gel system (reservoir-type patch), when heating of the formulation was not involved [24]. Accordingly, such reservoir-type patches, as well as topical formulations (solutions, gels) containing DMSO showed an enhanced permeation behavior [11,24,25]. The amount of DMSO incorporated in both topical formulations and reservoir type patches was able to establish its enhancing effect as no heat was applied during production, limiting the risk of DMSO evaporation.

Although DMSO is an excellent permeation enhancing agent, it needs to be mentioned that its administration can cause local skin irritations and a garlic-like breath due to its excretion via lungs that has been reported in some cases [11]. Nevertheless, the irritancy potential of a dermal formulation containing 45.5% of DMSO was clinically investigated and showed no adverse effects in most of the patients, while for the remaining cases mainly dry skin was reported [26]. Another study evaluated the relative irritancy of increasing DMSO concentrations in vivo and determined histopathological changes from 50% upwards [27]. Accordingly, as patches in the current study did not exceed 40% of DMSO content, one would only expect limited skin irritancy resulting from the new patch formulations.

Beside the significant permeation improvements, another advantage of the patches containing DMSO is the inhibition of E2 recrystallization, which is a common problem with actives such as estradiol. A higher drug load in the vehicle promotes a higher flux through the skin; therefore, supersaturation of transdermal systems is a widespread method to enhance transdermal performance. The problem is that supersaturated systems are only metastable. The drug is indeed at its highest thermodynamic activity, which enables a flux increased to a multiple; hence it also tends to form crystals, which reduces skin permeation [28]. The patches without DMSO showed crystals already after the production, which indicates a temporary existence of a supersaturated status. The patches with DMSO had the same drug load, but crystals were absent even after six months of storage. DMSO is not miscible with Duro-Tak^®^; the resulting matrix system of the patches could therefore be imagined as a homogeneous dispersion of DMSO in DT. Based on this assumption, a possible explanation for the stabilization could be the increased matrix solubility of E2 in the presence of DMSO, which inhibits the nucleation of E2 crystals in the matrix.

Drug permeation through the skin occurs in different steps. The first step is to release the drug from the patch matrix, followed by partitioning of the drug into the stratum corneum and diffusion through the stratum corneum. Permeating this primary barrier is the major rate-limiting step in the diffusion process of the drug through the skin, there upon the drug enters viable tissues, subsequently entering the systemic circulation. Considering the similar results of release studies and the missing correlation between permeation and release behavior of E2, it is obvious that the enhancement mechanism does not rely on a DMSO-supported faster release of E2 from the patch matrix. However, the flux of E2 increases significantly with the amount and flux of DMSO, which indicates a DMSO-dependent intradermal effect that translates into improvement of the skin permeation.

The increased drug flux is therefore a consequence of improving the second step of drug permeation, i.e., enhanced stratum corneum partitioning and diffusion. In order to allow simulating the partitioning between the different structures closest to the in vivo situation, full thickness skin was used in these experiments. Patches with a higher DMSO amount led to enhanced drug concentrations in the skin after permeation experiments. This observation points towards an increased drug permeation due to the enhanced solubility of E2 in the stratum corneum. It has been described earlier that a “push–pull effect” occurs when a faster permeating enhancer substance is added to the donor vehicle [29]. DMSO permeates faster into and through the stratum corneum than E2; in the skin, DMSO increases the drug solubility and a “pull effect” occurs, which favors the diffusion of the drug out of the donor vehicle. The correlation between flux of DMSO and E2 suggests that the permeation of E2 followed the permeation of DMSO closely. The excess free energy of E2 causes the “push effect” in the donor phase. DMSO permeation increases quickly at the beginning of the experiments and results in a steady-state plateau. This observation suggests that DMSO is able to build a reservoir in the skin providing enhanced solubility of E2. It was reported that DMSO enhanced drug permeation in the skin by affecting the stratum corneum structure (altering protein structures and interaction with lipids) [30,31]. This suggests faster drug permeation but the lag-time did not shorten with the presence of DMSO; thus, this mechanism is negligible. A further possible mechanism is the co-permeation of DMSO and the drug.

The delivered dose rate of the produced patches per day is comparable to the performance of two approved E2 matrix patches: Climara^®^ and Menorest ^®^ (both 50 µg/24 h for 7 days resp. 3–4 days of suggested use) [4]. Both patches have a larger size (13–15 cm^2^) and a higher drug load (4 mg) compared to our prepared patches (1.04 cm^2^, 0.7 mg). Due to the fact that after 28 h of permeation more than 80% of the drug still remains in our patches, a longer period of administration with constant drug levels is possible.

## 5. Conclusions

Integrating DMSO in transdermal patches exhibited several benefits compared to conventional patch formulations. The presence of DMSO within patch matrix strongly inhibited the recrystallization of E2 combined with a significant enhancement of drug permeation through the skin. The proportionality between DMSO content and drug flux surely needs to be confirmed for other actives however opens the option of tailor-made permeation kinetics based on the DMSO amount formulated in the patch. The largest drawback by now is the insufficient DMSO retention during storage that directly impacts the permeation and needs to be overcome in the next steps.

## Figures and Tables

**Table 1:** Exemplary compositions of the adhesive mass at different production times. From an initially (t = 0 h) viscous and liquid mixture to a homogeneous drug-in-adhesive layer (per DIA). Control was prepared without dimethyl sulfoxide (DMSO) (mean ± SD; n.d. = not detected).

Drying Conditions	0 h	20 °C, 4 h	35 °C, 3 h	40 °C, 2 h	35 °C, 4 h	40 °C, 3 h	50 °C, 24 h	Control 50 °C, 24 h
DMSO [%]	38.4 ± 2.2	62.9 ± 11.7	36.4 ± 3.8	29.5 ± 3.9	19.7 ± 1.7	6.9 ± 1.3	n.d.	-
EA [%]	42.5 ± 0.9	0.8 ± 0.0	n.d.	n.d.	n.d.	n.d.	n.d	n.d
Hexane [%]	2.1 ± 0.1	n.d.	n.d.	n.d.	n.d.	n.d.	n.d	n.d
Duro-Tak^®^ (solid content) [%]	16.2 ± 2.7	34.6 ± 4.3	48.8 ± 12.3	67.1 ± 10.1	76.3 ± 12.4	89.4 ± 9.4	95.6 ± 9.4	95.6 ± 7.1
ß-Estradiol [%]	0.8 ± 0.1	1.6 ± 0.8	2.4 ± 0.3	3.4 ± 0.5	4.0 ± 0.6	3.8 ± 0.6	4.4 ± 0.6	4.4 ± 0.5
Matrix weight [mg]	102.0 ± 0.8	57.0 ± 9.1	23.6 ± 2.0	20.7 ± 0.3	17.7 ± 1.8	15.8 ± 0.6	17.8 ± 1.1	18.3 ± 1.0
Appearance	Viscous liquid	Wet layer	Uniform DIA	Uniform DIA	Uniform DIA	Uniform DIA	Uniform DIA	Uniform DIA

**Table 2:** Flux values, lag time, mean delivered dose and kinetic release rate of the prepared patches.

Drying Conditions	35 °C, 3 h	40 °C, 2 h	35 °C, 4 h	40 °C, 3 h	50 °C, 24 h	Control 50 °C, 24 h
Flux J_ss_ E2 [µg/cm^−2^·h^−1^]	4.12 ± 0.3	3.2 ± 0.4	2.3 ± 0.2	1.8 ± 0.1	1.22 ± 0.2	1.1 ± 0.2
Lag-time [h]	5	5	7	6	3	3
Mean permeated dose [µg/24 h]	82	63	43	36	26	24
Kinetic release rate	1.61–1.70	1.65–1.69	1.68–1.73	1.58–1.66	1.42–1.52	1.52–1.58

## Data Availability

Not applicable.
